# Brain physiology during photoperiod-related caste determination in the primitively eusocial wasp *Polistes jokahamae*

**DOI:** 10.1038/s41598-024-80745-z

**Published:** 2024-12-05

**Authors:** Ken Sasaki, Hideto Yoshimura, Kakeru Yokoi

**Affiliations:** 1 Graduate School of Agriculture, Tamagawa University, Machida, Tokyo Japan; 2 Division of Crop Rotation Research for Lowland Farming, Tohoku Agricultural Research Center, NARO, Morioka, Iwate Japan; 3 Insect Design Technology Group, Division of Insect Advanced Technology, Institute of Agrobiological Sciences, National Agriculture and Food Research Organization (NARO), 1-2 Owashi, Tsukuba, 305-8634 Ibaraki Japan

**Keywords:** Entomology, Animal behaviour, Social evolution

## Abstract

Adult females of the primitively eusocial wasp *Polistes jokahamae* can change caste in response to photoperiod length. Short-day photoperiod females store more lipids but have less developed ovaries for hibernation (becoming gynes), whereas long-day photoperiod females have fewer lipid stores but more developed ovaries under queenless conditions (becoming egg-laying workers). To explore the physiological mechanisms underlying photoperiod-related caste determination, analyses of gene expression levels in the brains of short- or long-day females were performed. Results suggest that short-day females may exhibit higher expression of genes involved in tryptophan metabolism, insulin signaling, and nutrition, including the digestion of sugars and lipids, and production of royal jelly proteins. Oral administration of tryptophan resulted in a positive correlation between tryptophan levels in the brain and lipid stores in the abdomen, suggesting that tryptophan promotes lipid storage in gynes. Long-day females showed enhanced expression of genes involved in tyramine/dopamine syntheses, epidermal growth factor receptor, insulin degradation, and oogenesis. In these females, tyramine administration increased the brain levels of tyramine and dopamine, and activated initial ovarian development under queenless conditions. Together, these results indicate that photoperiod-related caste determination in *P. jokahamae* may involve gene expression pathways similar to those involved in caste determination during the preimaginal stage in other *Polistes* species.

**Supplementary Information:**

The online version contains supplementary material available at 10.1038/s41598-024-80745-z.

## Introduction

The reproductive division of labor in insects is a eusocial system enabling efficient colony growth and the production of reproductive individuals in the colony. A reproductive caste (queen or gyne) specializes in mating and egg-laying, and has a larger body size and longer lifespan compared with the nonreproductive caste (worker). The degree of specialization in each caste in terms of their morphology and behavior depends on how the colony is founded, whether the queens undertake worker-like tasks, and the level of eusociality^1–5^. In temperate eusocial species that hibernate, such as paper wasps, gynes have a large body size and considerable lipid stores, whereas in those species that undergo dependent colony founding (such as honey bees), including swarming and budding, queens show morphological specializations because they are not required to perform worker-like tasks when founding colonies^6^.

In temperate *Polistes* paper wasps, many species with independent colony founding produce workers during the colony growth stage in spring and summer, and then produce males and gynes during the mature colony stage in the autumn. The caste system in temperate *Polistes* wasps is initially influenced by nutrition, with vibration and photoperiod stimuli during the preimaginal stages^7–13^. It is ultimately determined by environmental stimuli, including the photoperiod, temperature, and colony conditions during the adult stage^14–18^, with the influence of caste-related physiology developing during the preimaginal stages. Therefore, temperate *Polistes* species can show caste plasticity in response to environmental factors during the adult stage. To do so, the brain is likely to have a crucial role in detecting environmental conditions for caste determination and for triggering changes in caste-specific characteristics, including behavior, lipid storage, and ovarian development. Clarification of these mechanisms would improve current understanding of how plasticity in paper wasps results in caste changes during the adult stage, and whether the wasps repurpose existing physiological mechanisms to determine castes in response to environmental stimuli.

In *Polistes jokahamae*, females decide their caste fate in response to the photoperiod^18^. The females store lipid for hibernation under short-day conditions and become gynes, whereas they develop ovaries without lipid storage under long-day conditions. The latter females with less lipid storage do not mate, do not hibernate, and therefore do not become founding queens the following spring, but they can lay unfertilized eggs as egg-laying workers under queenless conditions^18^. Gyne-destined females under short-day conditions have higher tryptophan levels in the brains compared with the worker-destined females^19^. Tryptophan is a precursor of indoleamines including serotonin and melatonin^20^. Serotonin modulates light-evoked responses in compound eyes^21^ and behaviors related to the circadian clock in insects^22,23^. Serotonin and melatonin control photoperiodic time measurements and diapause determination in insects^20^. Therefore, tryptophan metabolism is potentially linked to physiological responses related to photoperiod. Furthermore, egg-laying worker-destined females under longer-day conditions have higher tyramine and dopamine levels in the brains compared with gyne-destined females. Tyramine and dopamine are neuroactive substances and associated with transition to reproductive workers from normal workers in eusocial bees^24,25^. Thus, there are several candidate substances involved in caste-fate determination in response to photoperiod, but the effects of these substances on caste-fate determination in the brain have not yet been tested.

Physiological processes determining caste fate might be complex networks that activate lipid storage and mating for gynes or ovarian development for egg-laying workers. Transcriptome analysis using RNA-sequence (RNA-seq) is a powerful way to obtain comprehensive insights into caste-fate determination. To clarify the mechanism underlying photoperiod-related caste determination in the paper wasp, we first compared the gene expression pattern between gyne-destined and egg-laying workers-destined females following photoperiod manipulation. We also assayed the functions of candidate substances for promoting lipid store or ovarian activation. We hypothesized that tryptophan in the brain triggers the physiological processes involved in lipid stores, whereas tyramine in the brain activates ovarian development without lipid storage. We tested this hypothesis by investigating the effects of the oral application of tryptophan or tyramine on lipid stores and ovarian development in female paper wasps.

## Results

### Transcriptome analysis using females exposed to short- or long-day photoperiods

**Fig. 1:**
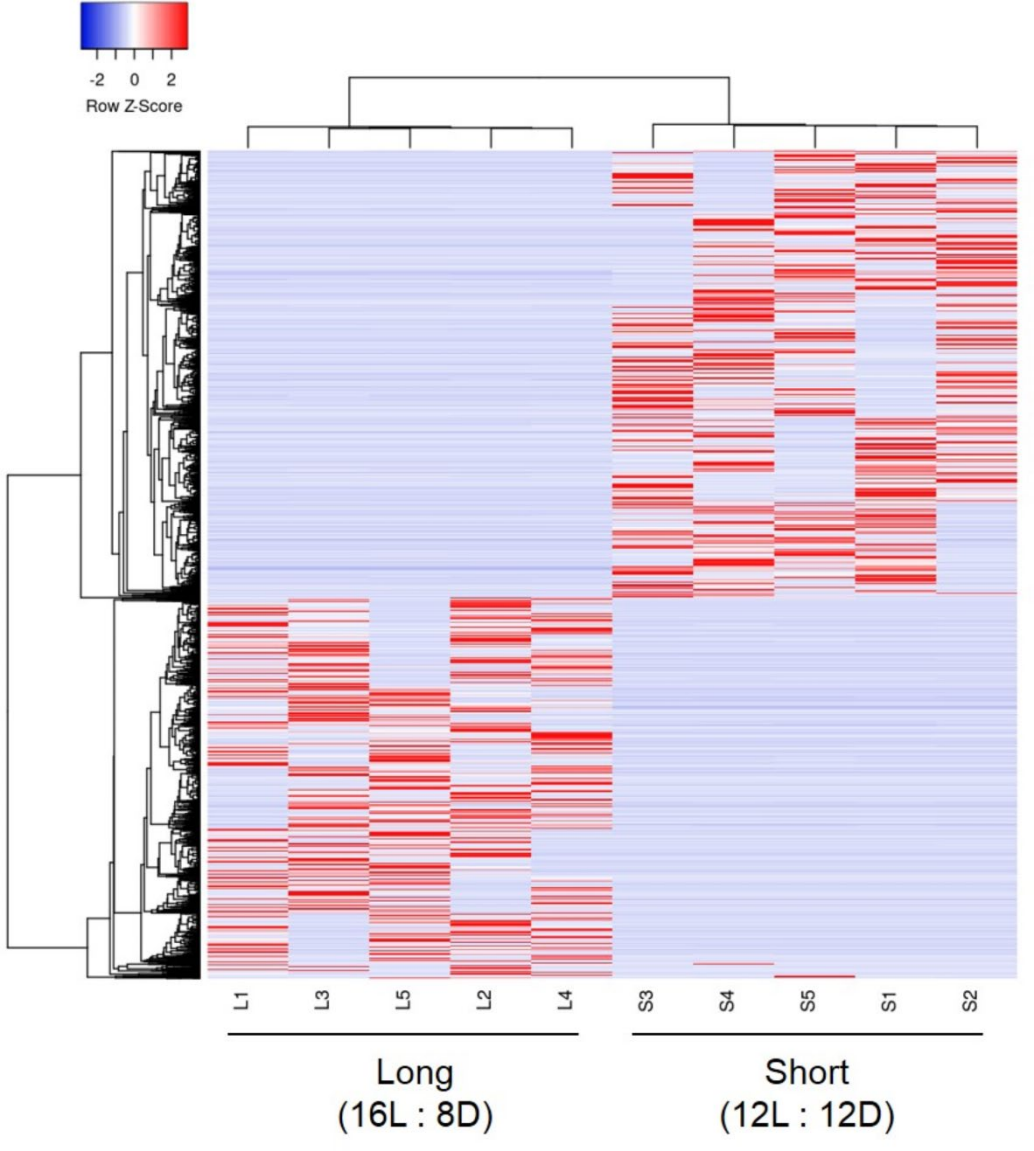
Differential gene expression in the brains of *Polistes jokahamae* females kept under long-day (Long) and short-day (Short) photoperiods analyzed by RNA-seq. Heat map of differentially expressed genes based on *p*-values (*p* < 0.05) in Long and Short females. Expression levels increased from blue to red. The letters at the bottom of the figure (L1–L5 and S1–S5) indicate the sample numbers (Long 1–Long 5 and Short 1–Short 5).

RNA-seq was performed to compare the expression levels of various genes in the brain of female wasps exposed to different photoperiod treatments (long-day versus short-day females). Approximately 40–57 million raw reads were generated from cDNA libraries, which were then used to construct ~ 344 thousand contigs by *de novo* assembly. The basal statistics of the contigs are shown in Supplemental Data 1 (DOI: 10.6084/m9.figshare.25864930). In total, 19,626 differentially expressed genes based on *p*-values (*p* < 0.05) were detected in a comparison between short- and long-day females (Supplemental Data 1), instead of *q*-values, because minimal *q*-values of these genes were 0.399 (see discussion). Of these, higher expression was detected in 10,437 genes in short-day females compared with 9189 genes in long-day females (Supplemental Data 1). These genes showed clear separation between photoperiod treatments and no overlap with individual variation (Fig. 1).

In the brains of short-day females, 823 upregulated genes were functionally annotated by blastx and defined locus names (Supplemental Data 1). Two of these genes encoded tryptophan 5-hydroxylase, involved in the synthesis of serotonin from tryptophan, and aromatic-L-amino acids decarboxylase, involved in monoamine synthesis (Table 1). Four genes were ecdysone- or juvenile hormone-induced genes, including Krüppel-like genes (Table 1). Thirteen genes were nutrition-related genes, including those encoding insulin-like peptide receptor, and genes involved in sugar and lipid metabolism, and major royal jelly protein production (Table 1). Two other genes were sex determination-related genes: *feminizer* and *doublesex* (Table 1).

In long-day females, 1281 upregulated genes were functionally annotated by Blastx search (Supplemental Data 1). Of these, two were genes encoding aromatic-L-amino acids decarboxylase having a different sequence from the gene in short-day females, and *N*-acetyltransferase, involved in monoamine metabolism (Table 2). A gene encoding epidermal growth factor receptor gene was also detected. Three genes related to juvenile hormone and three nutrition-related genes, including insulin-degrading enzymes, were detected. Five genes were associated with oogenesis. Three sex determination-related genes, including *fruitless* and *feminizer* (of a different sequence compared with short-day genes; Table 1), were detected. A Circadian clock gene was also detected.

**Table 1:** Focal genes upregulated in females with short-day photoperiod treatment. *B* biogenic amine, *E* ecdysteroid, *I* insulin, *J* juvenile hormone, *L* lipid, *M* major royal protein, *Su* sugar, *sd* sex determination.

Gene_id	a.value	m.value	*p*.value	q value	Annotation	Group
TRINITY_DN31136_c2_g2	4.108	9.748	0.031	0.399	Aromatic-L-amino-acid decarboxylase isoform X2 [*Polistes dominula*]	B
TRINITY_DN53634_c0_g1	2.259	4.815	0.048	0.399	Tryptophan 5-hydroxylase 1 [*Harpegnathos saltator*]	B
TRINITY_DN11535_c0_g1	−10.098	12.146	0.012	0.399	Ecdysone-induced protein 74EF isoform A isoform X1 [*Polistes canadensis*]	E
TRINITY_DN31073_c11_g1	4.663	8.290	0.010	0.399	Krüeppel-like factor 7 [*Habropoda laboriosa*]	J
TRINITY_DN30711_c0_g3	3.004	8.609	0.022	0.399	Krüeppel-like [*Polistes canadensis*]	J
TRINITY_DN31162_c6_g1	3.137	8.875	0.035	0.399	Krüeppel-like [*Polistes canadensis*]	J
TRINITY_DN23715_c0_g2	5.202	9.429	0.016	0.399	Isthmin-like [*Polistes canadensis*]	I
TRINITY_DN52681_c0_g1	4.734	10.894	0.020	0.399	Insulin-like peptide receptor [*Polistes canadensis*]	I
TRINITY_DN56384_c0_g1	4.766	8.861	0.012	0.399	UDP-glucose: glycoprotein glucosyltransferase isoform X2 [*Polistes dominula*]	Su
TRINITY_DN53166_c0_g1	3.002	6.578	0.034	0.399	Facilitated trehalose transporter Tret1-like [*Polistes dominula*]	Su
TRINITY_DN43671_c0_g1	3.778	8.257	0.043	0.399	Beta-glucuronidase-like [*Polistes canadensis*]	Su
TRINITY_DN45475_c0_g1	2.484	6.385	0.046	0.399	Fructose-1,6-bisphosphatase” 1 [*Polistes dominula*]	Su
TRINITY_DN29330_c15_g1	−1.210	10.836	0.010	0.399	Fatty-acid amide hydrolase 2-like isoform X2 [*Polistes canadensis*]	L
TRINITY_DN40046_c0_g1	5.024	9.169	0.012	0.399	Elongation of very long chain fatty acids protein AAEL008004-like [*Polistes dominula*]	L
TRINITY_DN64153_c0_g1	4.212	7.388	0.043	0.399	Putative fatty acyl-CoA reductase CG5065 [*Polistes canadensis*]	L
TRINITY_DN61671_c0_g1	−10.098	16.537	0.044	0.399	UDP-glucuronosyltransferase 1–8 [*Polistes dominula*]	L
TRINITY_DN23316_c0_g1	4.809	8.119	0.049	0.399	Pancreatic triacylglycerol lipase-like [*Polistes canadensis*]	L
TRINITY_DN66231_c0_g1	3.426	11.441	0.010	0.399	Major royal jelly protein 1 [*Polistes canadensis*]	M
TRINITY_DN26990_c0_g1	0.511	5.609	0.033	0.399	Yellow-like [*Polistes dominula*]	M
TRINITY_DN53366_c0_g1	−10.098	17.754	0.014	0.399	Fem-1 homolog B [*Polistes dominula*]	Sd
TRINITY_DN69096_c0_g1	6.166	7.644	0.025	0.399	Doublesex- and mab-3-related transcription factor A2-like [*Polistes canadensis*]	Sd

**Table 2:** Focal genes upregulated in females with long-day photoperiod treatment. *B* biogenic amine, *cc* circadian clock, *cd* caste determination, *I* insulin, *J* juvenile hormone, *L* lipid, *O* oogenesis, *sd* sex determination.

Gene_id	a.value	m.value	*p*.value	q value	Annotation	Group
TRINITY_DN31950_c3_g3	1.760	−6.334	0.048	0.399	Aromatic-L-amino-acid decarboxylase-like [*Polistes canadensis*], [*Polistes dominula*]	B
TRINITY_DN31699_c12_g2	3.124	−5.642	0.030	0.399	N-acetyltransferase 6 [*Polistes canadensis*]	B
TRINITY_DN29412_c5_g1	2.051	−5.406	0.024	0.399	Krüeppel-like factor 3 [*Polistes canadensis*]	J
TRINITY_DN30381_c0_g1	−10.098	−11.764	0.034	0.399	Venom carboxylesterase-6-like isoform X2 [*Polistes dominula*]	J
TRINITY_DN69172_c0_g1	−10.098	−12.681	0.029	0.399	Venom carboxylesterase-6-like [*Polistes canadensis*]	J
TRINITY_DN24914_c0_g2	4.282	−6.763	0.029	0.399	Epidermal growth factor receptor isoform X2 [*Polistes canadensis*]	Cd
TRINITY_DN32047_c4_g1	4.044	−8.725	0.027	0.399	Insulin-degrading enzyme isoform X2 [*Polistes canadensis*]	I
TRINITY_DN31881_c7_g5	2.416	−9.593	0.020	0.399	Niemann-Pick C1 protein-like [*Polistes dominula*]	L
TRINITY_DN33049_c3_g1	2.593	−9.714	0.021	0.399	stAR-related lipid transfer protein 3 isoform X1 [*Polistes dominula*]	L
TRINITY_DN29434_c4_g2	3.444	−9.416	0.011	0.399	Cueball [*Polistes canadensis*]	O
TRINITY_DN31008_c11_g1	4.013	−7.635	0.022	0.399	“Beta-1,4-mannosyltransferase” egh [*Polistes dominula*]	O
TRINITY_DN32794_c4_g1	1.667	−7.863	0.037	0.399	Chorion peroxidase isoform X2 [*Polistes dominula*]	O
TRINITY_DN30416_c9_g1	2.287	−5.955	0.044	0.399	Follistatin-A isoform X1 [*Polistes canadensis*]	O
TRINITY_DN29446_c6_g3	1.452	−7.273	0.048	0.399	Nuclear hormone receptor FTZ-F1 [*Polistes canadensis*]	O
TRINITY_DN32369_c2_g5	4.425	−5.887	0.027	0.399	Sex determination protein fruitless isoform X40 [*Orussus abietinus*]	Sd
TRINITY_DN30117_c5_g1	4.147	−6.837	0.044	0.399	Fem-1 homolog B-like [*Polistes canadensis*]	Sd
TRINITY_DN30675_c7_g2	2.568	−9.664	0.012	0.399	Virilizer isoform X1 [*Polistes canadensis*]	Sd
TRINITY_DN28339_c0_g1	4.261	−5.572	0.033	0.399	Pigment-dispersing hormone peptides (PDF) [*Camponotus floridanus*]	Cc

### Gene expression analyses by real-time quantitative PCR

**Fig. 2:**
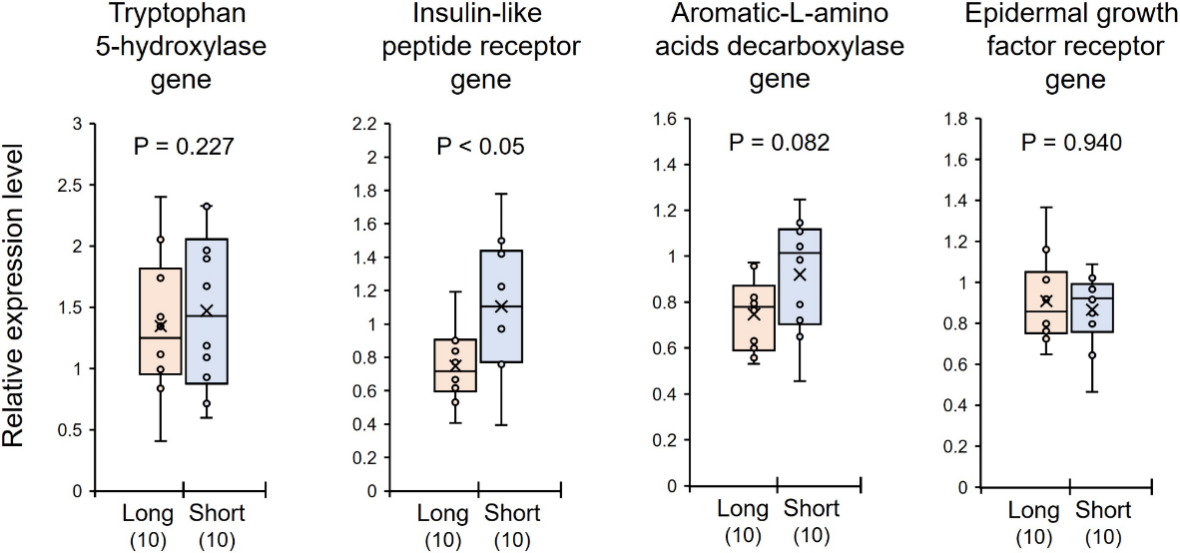
Relative gene expression in the brains between *Polistes jokahamae* females kept under long-day (Long) and short-day (Short) photoperiods in analyzed by RT-qPCR. The focal genes (tryptophan 5-hydroxylase gene, insulin-like peptide receptor gene, aromatic-L-amino acids decarboxylase gene and epidermal growth factor receptor gene) were examined. The small circles and “x” within the boxes indicate the distribution and mean of the data points, respectively. Numbers in parentheses indicate the number of samples examined. Significant differences were determined by Mann–Whitney* U* tests.

Quantification of gene expression was performed by real-time quantitative PCR (RT-qPCR) to confirm the expression of specific genes in the brain. Relative expression levels of four target genes (tryptophan 5-hydroxylase gene, insulin-like peptide receptor gene, aromatic-L-amino acids decarboxylase gene, and epidermal growth factor receptor gene) were quantified and compared between short- and long-day females. The expression levels of insulin-like peptide receptor gene were significantly higher in short-day compared with long-day females (Mann-Whitney U test, z = 2.192, *P* < 0.05, Fig. 2). By contrast, there was no significant difference in the relative expression levels of the other three genes between short- and long-day females (tryptophan 5-hydroxylase gene: z = 0.227, *P* = 0.821; aromatic-L-amino acids decarboxylase gene: z = 1.739, *P* = 0.082; epidermal growth factor receptor gene: z = 0.076, *P* = 0.940, Fig. 2).

### Lipid stores by tryptophan application

**Fig. 3:**
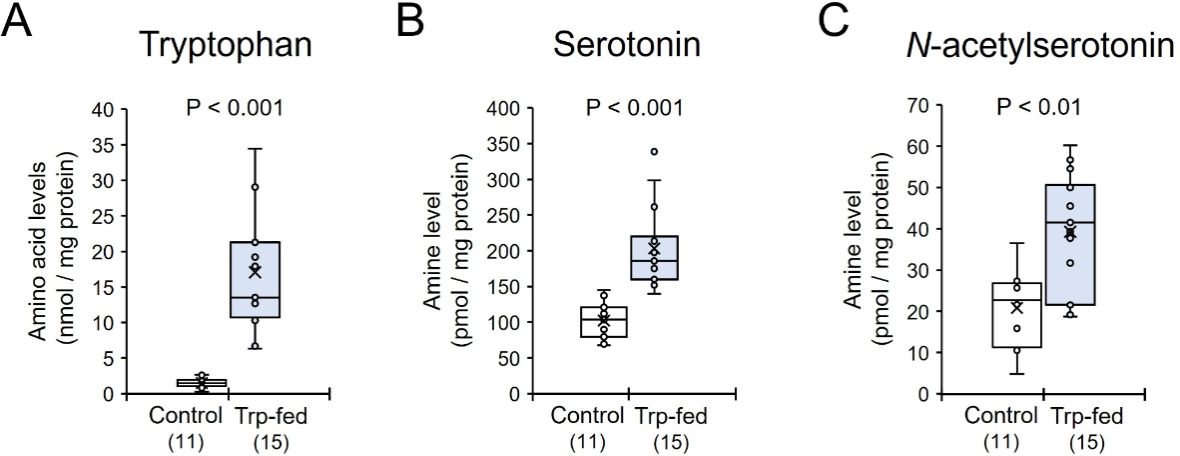
Levels of tryptophan (**A**), serotonin (**B**), and *N*-acetylserotonin (**C**) in the brains of tryptophan-fed (Trp-fed) or control *Polistes jokahamae* females. The small circles and “x” within the boxes indicate the distribution and mean of the data points, respectively. Numbers in parentheses indicate the number of samples examined. Significant differences were determined by Mann–Whitney* U* tests.

Tryptophan was applied orally to emerging isolated females (Tryp-fed) kept under a short-day photoperiod to test the effects of tryptophan on the induction of gynes with more lipid stores for hibernation. Oral application of tryptophan significantly enhanced the levels of tryptophan (Mann-Whitney U test, z = 4.282, *P* < 0.001, Fig. 3A), serotonin (a metabolite of tryptophan, z = 4.230, *P* < 0.001, Fig. 3B) and *N*-acetylserotonin (a metabolite of serotonin, z = 2.777, *P* < 0.01, Fig. 3C) in the brain of Trp-fed females compared with controls.

**Fig. 4:**
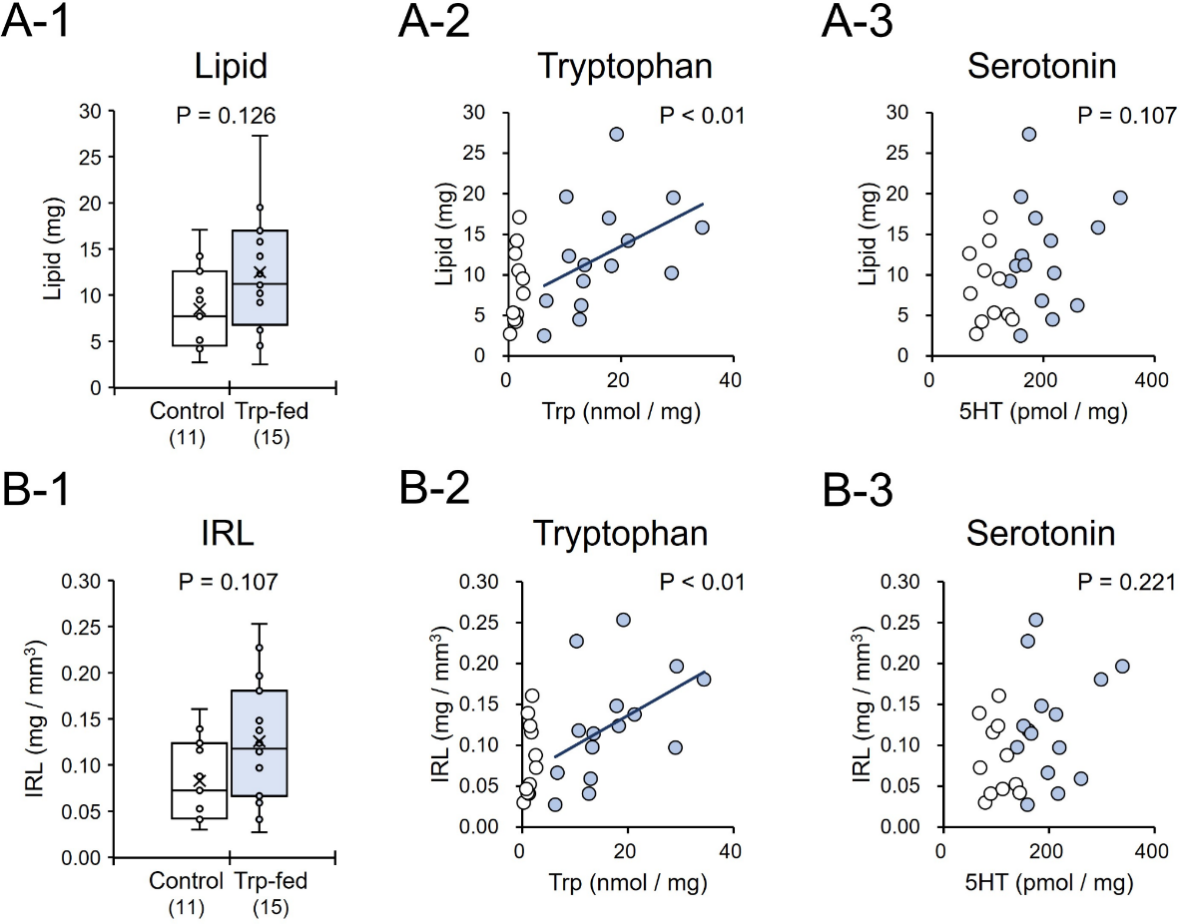
Levels of lipid stores and the index of relative lipid stores (IRL) in the abdomen of tryptophan-fed (Trp-fed) or control *Polistes jokahamae* females. (**A**) Comparisons of lipid stores between the two groups (A-1) and correlations between lipid stores and Trp (A-2) or serotonin (5HT) levels (A-3) in the brain. (**B**) Comparisons of IRL between the two groups (B-1) and between IRL and Trp levels (B-2) or 5HT levels (B-3) in the brain. In the boxplots (A-1 and B-1), the small circles and “x” within the boxes indicate the distribution and mean of the data points, respectively.

The levels of lipid stores in the abdomen did not significantly differ between Trp-fed and control wasps (Mann-Whitney U test, z = 1.531, *P* = 0.126, Fig. 4A-1). However, the levels of lipid stores were positively correlated with tryptophan levels in the brain (GLM, χ^2^ = 8.956, *P* < 0.01, Fig. 4A-2) but not with serotonin levels (χ^2^ = 2.598, *P* = 0.107, Fig. 4A-3). The index of relative lipid stores (IRL) did not differ significantly between Trp-fed and control wasps (Mann-Whitney U test, z = 1.614, *P* = 0.107, Fig. 4B-1). However, lRL was positively correlated with tryptophan levels in the brain (GLM, χ^2^ = 9.757, *P* < 0.01, Fig. 4B-2), but not with serotonin levels (χ^2^ = 1.501, *p* = 0.221, Fig. 4B-3).

### Ovarian activation by tyramine application

**Fig. 5:**
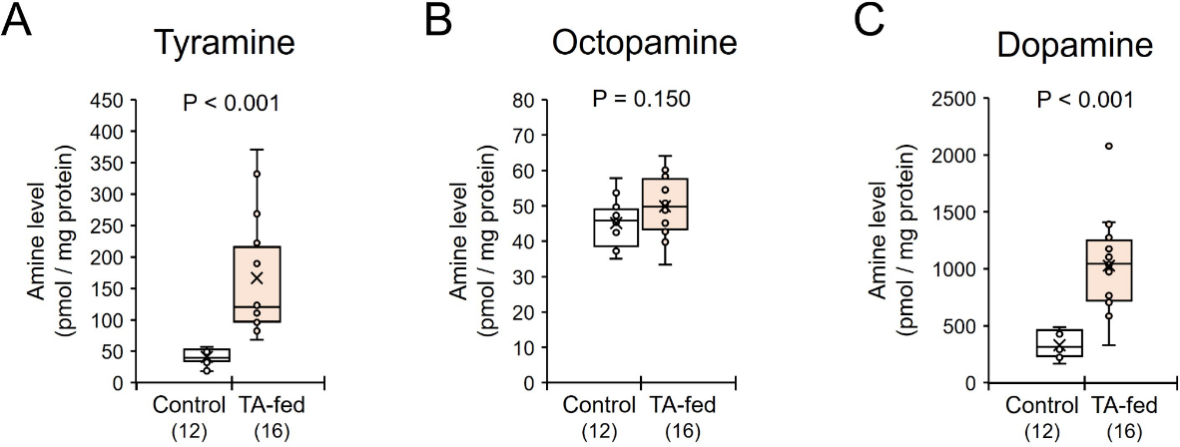
Levels of tyramine (**A**), octopamine (**B**), and dopamine (**C**) in the brains of tyramine-fed (Tyr-fed) or control *Polistes jokahamae* females. The small circles and “x” within the boxes indicate the distribution and mean of the data points, respectively. Numbers in parentheses indicate the number of samples examined. Significant differences were determined by Mann–Whitney *U* tests.

To determine the effects of tyramine on induction of egg-laying workers with developed ovaries, tyramine was orally applied to emerging isolated long-day females (TA-fed). TA-fed females had significantly enhanced levels of tyramine in their brains (Mann-Whitney* U* test, z = 4.457, *P* < 0.001, Fig. 5A), but not of octopamine (a metabolite of tyramine, z = 1.439, *P* = 0.150, Fig. 5B). Application of tyramine also significantly enhanced the levels of dopamine (z = 4.178, *P* < 0.001, Fig. 5C) in the brain.

**Fig. 6:**
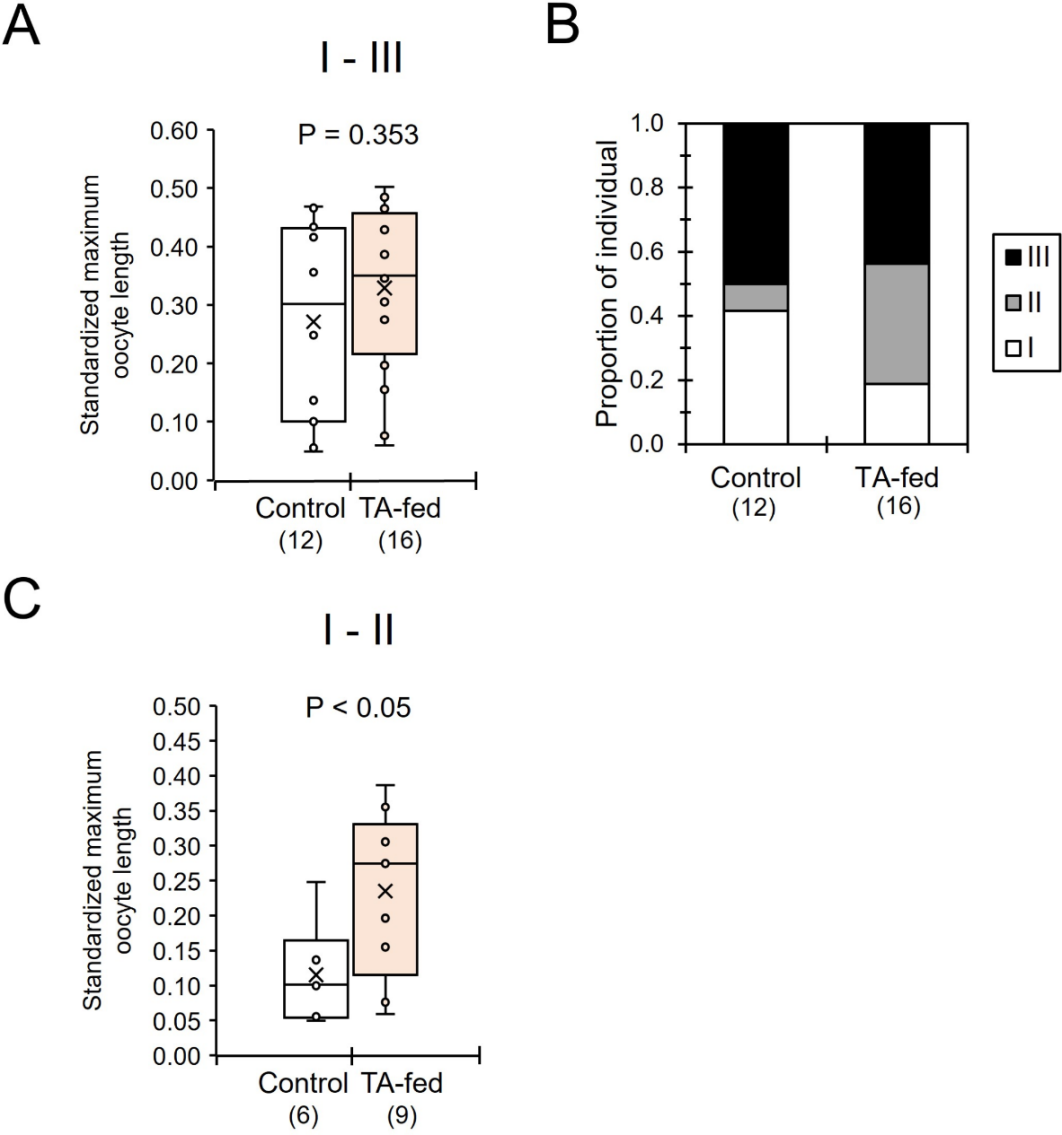
Ovarian development in tyramine (TA)-fed and control *Polistes jokahamae* females. (**A**) Comparisons of the standardized maximum oocyte length in ovaries by the head width between the two groups. (**B**) Comparisons of the proportion of females with different levels of ovarian development (levels I, II, and III). (**C**) Comparisons of the standardized maximum oocyte length in ovaries by the head width with level I and II development between the two groups. In the boxplots (**A**,**C**), the small circles and “x” within the boxes indicate the distribution and mean of the data points, respectively. Numbers in parentheses indicate the number of samples examined. Significant differences were determined by Mann–Whitney *U* tests.

When the maximum oocyte length was standardized by the head width as an indicator of the body size, the standardized oocyte length did not differ significantly between TA-fed and control individuals (Mann-Whitney *U* test, z = -0.928, *P* = 0.353, Fig. 6A). However, 44–50% of females in both groups had well-developed ovaries (level III) (Fig. 6B). Given that these individuals influenced the average maximum oocyte length in both groups, the data among individuals with undeveloped (level I) or middle-developed ovaries (level II) were also compared between TA-fed and control individuals. The standardized oocyte lengths by the head width among individuals with level I and II ovaries were significantly longer in TA-fed than in control individuals (z = 2.003, *P* < 0.05, Fig. 6C).

## Discussion

In eusocial hymenopterans, castes with distinct morphological characteristics are determined primarily by nutrition during the preimaginal stages^1–3,7,26^. However, in primitively eusocial wasps, the caste is initially influenced by nutrition and other stimuli during the preimaginal stages^7–13^ and then finally determined by environment stimuli during the adult stage^14–18^. The physiological mechanisms underlying caste determination during the preimaginal period have been investigated in several eusocial species of Hymenoptera^26^. However, the mechanism of caste determination during the adult stage has not been explored. Therefore, this study is the first attempt to investigate gene expression involved in photoperiod-related caste determination during the adult stage and test the effects of candidate substances on caste determination.

*Polistes* paper wasps habiting temperate regions show an ability to change caste or reproductive state in response to seasonal signals. In *P. jokahamae*, adult females respond to photoperiod length to become either gynes or workers^18^. Short-day females store more lipids for hibernation and have higher tryptophan levels in their brains, eventually becoming gynes; by contrast, long-day females store less lipid but have more developed ovaries with higher tyramine and dopamine levels in their brains, eventually becoming egg-laying workers^19^. The present study analyzed gene expression in the brains of short- and long-day females, and determined differentially expressed genes by RNA-seq and RT-qPCR under each photoperiod condition. We also tested the effects of tryptophan on lipid stores and effects of tyramine on ovarian activation, highlighting molecular mechanisms underlying photoperiod-related caste determination in *P. jokahamae.*

As revealed by RNA-seq, a specific gene encoding tryptophan 5-hydroxylase was detected as a differentially expressed gene based on *p*-value in the brains of females kept under a short-day photoperiod and, thus, destined to become gynes. Due to substantial individual variation in gene expression, the large number of assembled contigs, and limited sample size, the *q*-value for this gene was not significant, highlighting the need for further examination to confirm the findings. Tryptophan 5-hydroxylase mediates the synthesis of serotonin from tryptophan^27,28^, suggesting that tryptophan metabolism may be upregulated in gynes. The activation of tryptophan metabolism in gynes might result from the enhanced levels of tryptophan that occur in response to a short-day photoperiod^19^. Trp-fed females kept under a short-day photoperiod showed positive correlations between tryptophan levels in the brains and lipid stores or IRL levels in the abdomen, suggesting tryptophan as a key substance involved in induction of lipid storage for hibernation under a short-day photoperiod. However, there were no significant differences in lipid stores or IRL levels between Trp-fed and control individuals. Therefore, the effect of tryptophan on the induction of lipid storage under a short-day photoperiod may not be sufficient by itself and other factors may be required. Although Trp-fed females had significantly higher levels of tryptophan, serotonin, and *N*-acetylserotonin (a serotonin metabolite), lipid storage was associated with tryptophan, but not serotonin. In fact, there was no significant correlation between serotonin levels in the brains and lipid stores or IRL levels in the abdomen in Trp-fed wasps.

Nutrition-related genes encoding insulin-like peptide receptor, enzymes involved in sugar and lipid metabolism, and major royal jelly proteins were upregulated in the brains of short-day females. The higher expression of insulin-like signaling genes in gyne-destined females was also confirmed by RT-qPCR. Insulin-like signaling is a physiological pathway involved in caste determination during larval and pupal development in social Hymenoptera^26^. In adults, higher expression of insulin-related genes in the brains of gynes or queens have been reported in paper wasps^29,30^, ants^31^ and bumble bees^32,33^. In the paper wasp *Polistes metricus*, the expression level of genes encoding insulin-like peptide 2 (*PmILP2*) and insulin-like receptor 1 and 2 (*PmlnR1 and PmlnR2*) was higher in the brains of virgin gynes compared with workers^29,30^. A similar physiological brain state to the gynes in these species was recorded for *P. jokahamae* short-day females. This result provides insights into the plastic caste-fate determination mechanism during the adult stage in this species. Such photoperiod-related caste-fate determination might include common genes involved in caste determination during preimaginal stage in other *Polistes* species and maintain the common caste-specific states of gene expression.

Long-day females stored less lipids in their abdomens, but developed their ovaries under isolated conditions^18^. The brains of these females expressed genes encoding the epidermal growth factor receptor, insulin-degrading enzyme, Krüeppel-like factor, and oogenesis-related protein. In honey bees, suppression of the epidermal growth factor receptor gene expression by RNAi inhibited ovarian development in queenless workers^34^. In bumble bees, the expression of Krüeppel-like factors was enhanced by juvenile hormone, which activates ovarian development in workers^35^. Thus, the gene expression patterns recorded in the brains of long-day females might reflect involvement in ovarian activation and induction of worker traits.

Female *P. jokahamae* destined to become egg-laying workers following long-day photoperiod exposure express higher tyramine and dopamine levels compared with short-day females^19^. The higher expression level of the gene encoding aromatic-L-amino acids decarboxylase in long-day females might be related to tyramine synthesis by tyrosine decarboxylase and/or dopamine synthesis by DOPA decarboxylase. Tyramine administration to long-day females in the current study enhanced tyramine and dopamine levels in the brain and activated initial-stage ovarian development (level II). Therefore, tyramine and/or dopamine have functions that promote initial ovarian development. However, the maximum oocyte lengths among individuals with level I, II and III ovaries were not significantly different between TA-fed and control wasps. This might be due to the long isolation period (14 days) for ovarian development and the large proportion of individuals with well-developed ovaries. In *Polistes chinensis*, an effect of dopamine on ovarian development in workers isolated for 10 days was detected^36^. It is also possible that other factors promote ovarian development in isolated females in *P. jokahamae* under a long-day photoperiod, in parallel with the effects of tyramine and/or dopamine. A similar function of tyramine in ovarian activation was reported in queenless workers of the honey bee^25,37^. Dopamine function in ovarian activation also has been reported in workers of the paper wasp *P. chinensis*^36,38^, a honey bee^24,39,40^, and reproductive females in ants^41,42^. Thus, ovarian activation by tyramine/dopamine in workers might be a common feature among social hymenopterans, resulting in the induction of egg-laying workers under a long-day photoperiod.

## Conclusion

This study analyzed gene expression levels in the brains of female *P. jokahamae* in response to different photoperiod lengths. Oral applications of tryptophan or tyramine were also tested to determine whether these candidate substances induce lipid storage in gynes or ovarian activation in egg-laying workers. Females exposed to a short-day photoperiod showed enhanced expression of genes involved in tryptophan metabolism, insulin signaling, and nutrition, including the metabolism of sugars and lipids, and production of royal jelly proteins. Oral administration of tryptophan resulted in a positive correlation between tryptophan levels in the brain and lipid stores in the abdomen, suggesting that tryptophan promotes lipid storage in gyne-destined females. Long-day females showed enhanced expression of genes encoding epidermal growth factor receptor, and involved in insulin degradation and oogenesis, while oral tyramine increased the brain levels of tyramine and dopamine, and activated initial ovarian development. The gene expression patterns in each caste suggest that photoperiod-related caste determination in *P. jokahamae* is promoted by common caste determination genes during preimaginal stages in other *Polistes* species, and that the common caste-specific states of gene expression may be maintained.

## Materials and methods

### Experimental setup

Twelve colonies of *P. jokahamae* were collected from Morioka, Iwate, Japan. All adults were removed from the nests when they were collected in the field, and only nests with immature wasps, including pupae, were kept in individual small transparent plastic cups (129 mm in diameter × 58 mm high) in a temperature- and photoperiod-controlled room. The temperature was maintained at 25 ± 1 ºC and the photoperiod was set to simulate the natural changes of daylength (light: dark = 14 h:10 h) in Morioka (N39°42ʹ/E141°09ʹ). A total of 114 females emerging from 12 colonies collected during or after male emergence were used for the study (Supplementary data).

On emergence, each female were transferred to an individual small transparent plastic cup (129 mm in diameter × 58 mm high) containing honey, a larva of the silk moth (*Bombyx mori*), and water and kept under short-day photoperiod (light: dark = 12 h:12 h) or long-day photoperiod (light: dark = 16 h:8 h) for 14 days. The silk moth larva in each cup was replaced if it was injured by the wasp or died. After 14 days of isolation, all females were euthanized with liquid nitrogen. The frozen head was removed from the body, and kept in liquid nitrogen until RNA extraction. The gaster was stored in a freezer at − 20ºC and used to measure the lipid stores and oocyte length.

### RNA extraction

Frozen brains were dissected in ice-cold double-sterilized 0.1 M phosphate buffer (pH 7.0) on a Peltier cooling unit at ~ 4 °C covered with a flexible film (Parafilm, Bemis Company, Chicago, IL, USA) under a dissecting microscope. Two dissected brains with subesophageal ganglia were homogenized with an electric homogenizer (T10 + S10N-5G, IKA Works, Staufen, Germany) in extraction buffer from an ISOGEN kit (NipponGene, Tokyo, Japan). Total RNA was extracted from the two brains using an ISOGEN RNA isolation kit according to the manufacturer’s instructions. During RNA extraction, RNA was treated with rDNase (RT Grade for Heat Stop, Nippongene) for 15 min to remove genomic DNA and then mixed with stop solution at 70 °C for 10 min. The quality and quantity of extracted RNA were determined at 230, 260, and 280 nm using a microvolume spectrophotometer (Nanodrop 2000, Thermo Fisher Scientific, Waltham, MA, USA). Each of five samples contained the RNA from the brains of two females kept under short- or long-day photoperiods were used for RNA-seq. Ten females from each of the three colonies were equally divided between short- and long-day photoperiod conditions.

### cDNA library preparation and sequencing

Before preparing cDNA libraries, the total RNA integrity was checked by using an Agilent 2100 Bioanalyzer (Agilent Technologies, Santa Clara, CA, USA). Using the TruSeq Standard mRNA LT Sample Prep Kit (Illumina, San Diego, CA, USA), a library was constructed from total RNA according to the manufacturer’s protocol. RNA-seq was performed on the RNA samples using Illumina Novaseq 6000 platform (Illumina) by Macrogen Japan (Kyoto, Japan). The raw sequence data were deposited in the DNA Data Bank of Japan (DDBJ) Sequence Read Archives (DRA) (accession numbers: DRR550274 (Long-day photoperiod brain sample biological replicate 1 (L-1)), DRR550275 (L-2), DRR550276 (L-3), DRR550277 (L-4), DRR550278 (L-5), DRR550279 (Short-day photoperiod brain sample biological replicate 1 (S-1)), DRR550280 (S-2), DRR550281 (S-3),DRR550282 (S-4), DRR550283 (S-5)).

### Constructions of reference contigs

All the raw reads were quality controlled and adapter sequences were removed by Trim Galore version 0.6.10 (https://github.com/FelixKrueger/TrimGalore). Reference contigs were constructed by Trinity version 2.11.0^43^ using the trimmed sequence data. The reference contig data were deposited in the Transcriptome Shotgun Assembly (TSA) database (accession numbers: ICWH01000001- ICWH01344170, List of IDs of Trinity and TSA was uploaded in Supplemental Data 2, DOI: 10.6084/m9.figshare.25879945). A ‘Gene_id’ was allocated to each contig by Trinity software.

Each contig was annotated following Uchibori-Asano et al.^44^. Briefly, the contig sequence data were analyzed as queries using blastx (e-value < 1e^− 3^) against the National Center for Biotechnology Information non-redundant (NCBI-nr) protein database and a top-hit description was adopted as an annotation for each contig.

### Analysis of differentially expressed genes

Transcripts per million (TPM), mapped tag counts as ‘expected_count’ (not normalized), were calculated for each contig by RSEM version 1.2.7 operated by ‘align_and_estimate_abundance.pl’ in the Trinity package version Trinity r20140717^43,45^ (The output files of ‘align_and_estimate_abundance.pl’ of each sample including TPM, expected_count, were uploaded in Supplemental Data 3, DOI: 10.6084/m9.figshare.25880020). For the detection of differentially expressed genes between castes, iDEGES/edgeR in the TCC package version1.8.2 was used with *p-value* < 0.05, and fold changes of normalized tag count > 2 with ‘expected_count data’^46^ by using gene level count tag data (Output file of iDEGES/edgeR is available in Supplemental Data 4, DOI: 10.6084/m9.figshare.25880137). Essentially, both *p-* and *q*-values indicate the degree of difference in the expression levels of genes. While *p*-values test for initial statistical significance, *q*-values adjust for multiple comparisons to control the false discovery rate (FDR). In situations where the physiological state within a group is homogenous and less variable, *q*-values are typically preferred for identifying differentially expressed genes because they help reduce the risk of false positives in large datasets^47,48^. However, in datasets with high individual variability and large numbers of comparisons, as in the current study, where field-collected paper wasps showed considerable physiological variability and approximately 344,000 contigs were assembled, *q*-values can be inflated, leading to very few or no genes passing the significance threshold after adjustment (Tables 1 and 2). In these cases, although false positive differentially expressed genes may be included, *p*-values can be used to determine the degree of difference in gene expression levels, which can be useful for further validation experiments. Therefore, *p*-values were used to determine the degree of difference in gene expression levels.

### Measurements of relative expression levels of focal genes in the brain by RT-qPCR

RT-qPCR analyses were performed on the focal genes to confirm the differential gene expression between short- and long-day females revealed by RNA-seq. RNA extraction was conducted by the same procedure used for RNA-seq from two brains from females from the same treatment group. For single-strand cDNA synthesis, DNase-treated RNA (500 ng) was transcribed using a high-capacity cDNA Reverse Transcription kit (Applied Biosystems, Waltham, MA, USA) according to the manufacturer’s instructions. Negative control samples without reverse transcriptase were treated using the same procedure. Ten RNA samples from each group, with each sample containing the brains from two females kept under either short- or long-day photoperiods were examined. Twenty females from each of the three colonies were divided between short- and long-day photoperiod conditions.

Four genes (tryptophan 5-hydroxylase gene, insulin-like peptide receptor gene, aromatic-L-amino acids decarboxylase gene and epidermal growth factor receptor gene) were selected as target genes for RT-qPCR analyses (Table S1). Genes encoding tryptophan 5-hydroxylase and aromatic-L-amino acid decarboxylase were associated with the synthetic and metabolic pathways of tryptophan and tyramine, respectively. Insulin-like peptide receptor gene and epidermal growth factor receptor gene have been known as genes involved in queen production during pre-imaginal stages in other species^26^ and transition to egg-laying workers in honey bees^34^. Since these genes were hit as differentially expressed genes in RNA-seq, they were selected as target genes for RT-qPCR analyses. Three reference genes (40 S ribosomal protein S3 and S5 genes, and 60 S ribosomal protein gene) were examined with sets of primers (Table S1). The primer sequences of target and reference genes were designed using Primer 3 Plus (www.bioinformatics.nl/cgi-bin/primer3plus/primer3plus.cgi). Standard regression lines were generated for each target and reference gene (at 1, 1/10, 1/20, and 1/40 dilutions) and based on the relative concentration of cDNA and the quantification cycle (Cq). The cDNAs from short-day females were used as a RT-qPCR template. RT-qPCR was performed followed the method described by Sasaki et al.^33^. An individual sample was repeated three times in a single run of the RT-qPCR. Amplification of the single product was confirmed by dissociation curve analysis using a real-time PCR system.

To estimate the mRNA expression levels of each target gene, we recorded the Cq values of the reference and target genes. The suitability of three reference genes as internal control genes was evaluated by a Mann–Whitney *U* test. The Cq values of 40 S ribosomal protein S3 gene were the most stable and not significantly different between castes (Mann–Whitney U test, N_long−day_ = N_short−day_ = 10, 40 S ribosomal protein S3 gene: z = 1.209, *P* = 0.227; 40 S ribosomal protein S5 gene: z = 1.285, *P* = 0.200; 60 S ribosomal protein gene: z = 1.436, *P* = 0.151). Therefore, we normalized the expression levels of target genes by using expression levels of 40 S ribosomal protein S3 gene. These analyses were performed with reference to the Minimum Information for Publication of Quantitative Real-Time PCR Experiments guidelines^49^.

### Application of drugs

Gyne-destined females under short-day conditions have higher tryptophan levels in the brains compared with the worker-destined females, whereas egg-laying worker-destined females under longer-day conditions have higher tyramine and dopamine levels in the brains compared with gyne-destined females^19^.

To test whether tryptophan could induce females to become gynes with more lipid stores for hibernation, each newly emerging female was transferred to an individual plastic cup (129 mm in diameter × 58 mm high) and provided with 1 mg/mL tryptophan dissolved in 30% sucrose solution for 14 days under a short-day photoperiod (Trp-fed). The concentration of tryptophan was based on the effective concentration in experiments of oral intake of biogenic amine precursors^40,50^ Control females were also reared individually and provided only 30% sucrose solution under a short-day photoperiod for 14 days. Fifteen and eleven females from five colonies were examined in Trp-fed and control groups, respectively (Supplementary data).

To test whether tyramine could induce females to become egg-laying workers with developed ovaries, newly emerging females were each isolated as described above and provided with 1 mg/mL tyramine dissolved in 30% sucrose solution for 14 days under a long-day photoperiod (TA-fed). The concentration of tyramine was based on the effective concentration in experiments of oral intake of biogenic amines^36,51^. Control females were also reared individually and provided only 30% sucrose solution under a long-day photoperiod. Sixteen and twelve females from five colonies were examined in TA-fed and control groups, respectively (Supplementary data).

Neither oral treatment influenced the survival of the females, with all tested individuals surviving. After 14 days of isolation, females were euthanized with liquid nitrogen and their heads were stored in liquid nitrogen until quantification of biogenic amines. The gaster was stored in the freezer at − 20ºC for measurement of oocyte length and lipid stores.

### Measurements of biogenic amines

Each sample was prepared for high-performance liquid chromatography-electrochemical detection (HPLC-ECD) analysis based on Yoshimura et al.^19^. Frozen brains with subesophageal ganglia were dissected out and homogenized in 100 µL of ice-cold 0.1 M perchloric acid containing 12.5 ng/mL 3,4-dihydroxybenzylamine (DHBA, as the internal standard) for 2 min. Each sample was transferred to a 1.5 mL Eppendorf tube and then centrifuged at 15,000×*g* for 30 min at 0 ºC. Supernatants were then transferred to a microvial for HPLC-ECD analysis.

The HPLC-ECD system and mobile phase mostly conformed to the methods described by Yoshimura et al.^19^. Briefly, the system comprised a solvent delivery pump (PU-2080, JASCO, Tokyo, Japan), a refrigerated automatic injector (AS-2057, JASCO), a C_18_ reverse-phase column (250 × 4.6 mm i.d., 5 μm average particle, Capcell Pak UG120, Shiseido, Tokyo, Japan) maintained at 35 °C in a column oven, and an electrochemical detector (ECD-700, EICOM, Kyoto, Japan) set at 0.8 V under 35 ºC. The mobile phase comprised sodium-1-octanesulfonate (1.62 mM) and 5.3% acetonitrile with 0.18 M monochloroacetic acid and 40 µM Na _2_-EDTA adjusted to pH 3.6 with NaOH, which maintained a flow rate at 0.7 mL/min. External standards, including tyramine, octopamine, dopamine, tryptophan, serotonin, *N*-acetylserotonin, and DHBA, were run before and after the sample runs to identify and quantify biogenic amines in the brain. The biogenic amine peaks of samples were identified by retention time and hydrodynamic voltammograms compared with those of external standards using PowerChrom version 2.5 software (eDAQ Pty Ltd., Sydney, Australia). Each value of a biogenic amine was quantified by calculating the ratio of the peak area of a focal biogenic amine in the sample to that of the external standard.

To standardize the levels of biogenic amines in the brain based on the protein content, the amount of protein in the brain was quantified using the Bradford method^52^. The method described in Yoshimura et al.^19^ was applied to the residues treated with 0.1 M perchloric acid. These residues were neutralized with 50 µL 0.5 M NaOH. After ultrasonic dissolution of the residues for 15 min, the solution was diluted with 200 µL 0.1 M phosphate buffer (pH 7.0). Bovine serum albumin was used as a standard and was dissolved in NaOH: phosphate buffer (1:4) to 1/10, 1/20, 1/40, and 1/80 dilutions. The samples and standard solutions were reacted with a protein assay stain (500-0006, Bio-Rad, Hercules, CA, USA) for 5 min in a 96 well-plate, and their absorption was measured on a microplate reader (SH-1200Lab, Corona Electric, Ibaraki, Japan) with a 595 nm wavelength. Protein content was calculated using a calibration curve of the standard solutions.

### Evaluation of ovarian development

Ovarian activity was evaluated by measuring the length of the largest terminal (basal) oocytes and classifying the stage of the most-activated oocyte in the ovarioles. The maximum oocyte length was standardized by the head width as an indicator of the body size. The widest part of the head of newly emerged females was measured as a head width using a caliper with a precision of 0.01 mm. Three ovarian stages were defined based on the size and shape of oocyte and trophocyte chambers, based on the following criteria: (1) stage 1: ovaries were clearly distinguishable but had small (almost spherical) oocytes and a much larger trophocyte chamber (oval in shape) (largest terminal oocyte length ≤ 0.8 mm); (2) stage 2: oocytes larger than in stage 1 and elongated, but the trophocyte chamber was still larger than the oocyte; and (3) stage 3: oocytes now oval in shape and as large or larger than the trophocyte chamber. Each pair of ovaries was carefully removed from the abdomen under a dissecting microscope. Photographic images were taken with a HDCE-X3N digital microscope camera on a stereomicroscope (Leica S9D, Leica Microsystems, Wetzlar, Germany) and analyzed using ImageJ 1.53e (National Institutes of Health, Bethesda, MD, USA).

### Evaluation of lipid stores

Lipid stores in the gaster, including the ovary, were measured by calculating the difference in dry mass before and after lipid extraction by diethyl ether^53^. The removed ovaries were carefully returned for measurements of lipid stores. These processes were performed equally for all individuals so as not to affect the evaluation of lipid stores. Dry mass was measured by an electronic balance with a precision level of 0.0001 g.

### Statistical analyses

The data did not meet the criteria for parametric tests and, therefore, nonparametric tests were used for comparisons between two groups. The Mann–Whitney *U* test was used to compare the relative gene expression levels measured by RT-qPCR between photoperiod treatments. The levels of biogenic amines in drug-treated versus control groups were also compared using the Mann–Whitney *U* test. To confirm the correlation between lipid stores and the levels of amino acids or biogenic amines, a linear mixed model was conducted by using the ‘lme4’ package in R (version 3.6.0)^54^. Lipid stores were incorporated in the model as the response variable, and the levels of amino acids and biogenic amines as explanatory variables. Colony identity was incorporated as a random effect. The levels of tryptophan standardized (mean is 0, standard deviation is 1), because the data scale largely varied between 26 samples. Probability values for the statistical tests were calculated by using the likelihood ratio test using the ‘Anova’ function in the ‘car’ package in R. Lipid stores were controlled by the head width cubed (the index of relative lipid stores, IRL) as described by Yoshimura and Yamada^55^, and were logarithm transformed.

## Electronic supplementary material

Below is the link to the electronic supplementary material.

Supplementary Material 1

Supplementary Material 2

Supplementary Material 3

## Data Availability

The datasets generated and analyzed during the current study are available in the DNA Data Bank of Japan (DDBJ) Sequence Read Archives (DRA) (accession numbers: DRR550274–DRR550283), the Transcriptome Shotgun Assembly (TSA) database (accession numbers: ICWH01000001–ICWH01344170), and the Figshare (DOI: 10.6084/m9.figshare.c.7249951; DOI: 10.6084/m9.figshare.25864930; DOI: 10.6084/m9.figshare.25879945; DOI: 10.6084/m9.figshare.25880020; DOI: 10.6084/m9.figshare.25880137). Other data are available within the paper and its supplementary materials.
