# Supplementary material for: Brain physiology during photoperiod-related caste determination in the primitively eusocial wasp Polistes jokahamae
Source: Sci Rep. 2024 Dec 5;14:30399. doi: 10.1038/s41598-024-80745-z (PMC11621535; doi:10.1038/s41598-024-80745-z)
Supplement: Supplementary file 2 — Supplementary Material 2 [file 41598_2024_80745_MOESM2_ESM.pdf]

Table S1. Primer sequences of reference and target genes for RT-qPCR.

	Genes	Forward primer	Reverse primer
Reference genes			
TRINITY_DN29606_c5_g1_i2	40S ribosomal protein S3 gene	AGTTTGCCAACAGGATCCCA	CGAGTTCCTTACCCGTGAGC
TRINITY_DN32696_c1_g1_i1	40S ribosomal protein S5 gene	GAATGCTGCTTCTCGTGCAC	GGCCTTTCCTGTTTGTGCTC
TRINITY_DN33296_c0_g1_i4	60S ribosomal protein gene	GCTTTGGTAGTCCCTCTCTGAAT	ACAGCCAAAATGAGGAAGACC
Target genes			
TRINITY_DN53634_c0_g1	Tryptophan 5-hydroxylase 1 gene	ACTTCGACCAGTTGCAGGAT	AAAAGGATCCGAAGAATGACG
TRINITY_DN52681_c0_g1	Insulin-like peptide receptor gene	TGAGAGGGGAGAAGCAAGAA	CAGAGTAGCACGAGCAGCAG
TRINITY_DN31950_c3_g3	Aromatic-L-amino acids decarboxylase gene	TACCACCAATCGTTGCTTGA	AGTTGGCCGATATTCGTGAG
TRINITY_DN24914_c0_g2	Epidermal growth factor receptor gene	ATGGCGACTGGAATTTTGAC	AACGAACCACTTCGACCAAC
