# Supplementary material for: Brain physiology during photoperiod-related caste determination in the primitively eusocial wasp Polistes jokahamae
Source: Sci Rep. 2024 Dec 5;14:30399. doi: 10.1038/s41598-024-80745-z (PMC11621535; doi:10.1038/s41598-024-80745-z)
Supplement: Supplementary file 3 — Supplementary Material 3 [file 41598_2024_80745_MOESM3_ESM.docx]

**Supplemental Data**

All Supplemental Data are available in Figshare. DOI: 10.6084/m9.figshare.c.7249951

**Supplemental Data 1**

Up-regulated DEG lists in short-day photoperiod females and long-day photoperiod female brain samples. Short_up_DEG.xlsx and Long_up_DEG.xlsx contain up-regulated DEGs with normalized tag count value of each sample, several values related to expressions and functional annotations in short-day photoperiod females and long-day photoperiod female brain samples, respectively. Long_up_DEG_with_functional_annotation.xlsx and Short_up_DEG_with_functional_annotation.xlsx is the lists of the DEGs with functional annotations of long-day and short-day photoperiod female brain samples, respectively. DOI: 10.6084/m9.figshare.25864930

**Supplemental Data 2**

Trinity and TSA ID list. Trinity_TSA_ID_list contains Trinity and TSA IDs of each contig. DOI: 10.6084/m9.figshare.25879945

**Supplemental Data 3**

Output files of RSEM. L-vs-S.isoforms.matrix.tpm and L-vs-S.genes.matrix.tpm contain Tpm values in all samples at all contig level and gene level (allocated by Trinity software) respectively, while mapped tag counts values at all contig level and gene level are shown in L-vs-S.genes.matrix.counts and L-vs-S.isoforms.matrix.counts, respectively. DOI: 10.6084/m9.figshare.25880020

**Supplemental Data 4**

Output files of iDEGES/edgeR. DOI: 10.6084/m9.figshare.25880137
